# Sketch distance-based clustering of chromosomes for large genome database compression

**DOI:** 10.1186/s12864-019-6310-0

**Published:** 2019-12-30

**Authors:** Tao Tang, Yuansheng Liu, Buzhong Zhang, Benyue Su, Jinyan Li

**Affiliations:** 1 Advanced Analytics Institute, Faculty of Engineering and IT, University of Technology Sydney, Broadway, Sydney, NSW 2007 Australia; 2 School of Computer and Information, Anqing Normal University, Anqing, 246401 China

**Keywords:** NGS data, Data compression, Reference-based compression, Unsupervised learning

## Abstract

**Background:**

The rapid development of Next-Generation Sequencing technologies enables sequencing genomes with low cost. The dramatically increasing amount of sequencing data raised crucial needs for efficient compression algorithms. Reference-based compression algorithms have exhibited outstanding performance on compressing single genomes. However, for the more challenging and more useful problem of compressing a large collection of *n* genomes, straightforward application of these reference-based algorithms suffers a series of issues such as difficult reference selection and remarkable performance variation.

**Results:**

We propose an efficient clustering-based reference selection algorithm for reference-based compression within separate clusters of the *n* genomes. This method clusters the genomes into subsets of highly similar genomes using MinHash sketch distance, and uses the centroid sequence of each cluster as the reference genome for an outstanding reference-based compression of the remaining genomes in each cluster. A final reference is then selected from these reference genomes for the compression of the remaining reference genomes. Our method significantly improved the performance of the-state-of-art compression algorithms on large-scale human and rice genome databases containing thousands of genome sequences. The compression ratio gain can reach up to 20-30% in most cases for the datasets from NCBI, the 1000 Human Genomes Project and the 3000 Rice Genomes Project. The best improvement boosts the performance from 351.74 compression folds to 443.51 folds.

**Conclusions:**

The compression ratio of reference-based compression on large scale genome datasets can be improved via reference selection by applying appropriate data preprocessing and clustering methods. Our algorithm provides an efficient way to compress large genome database.

## Introduction

Next-generation sequencing (NGS) technologies have produced enormous amount of reads data at an unprecedented speed [1]. The sharp reduction in sequencing costs has also provoked a wide range of NGS applications in large scale health, environment, and agriculture genomic research. One example is the 1000 Genomes Project [2]. The NGS data generated by this project in the first six months exceeded the accumulated sequence data in NCBI during the past 21 years [3]. This project finished the sequencing of 1092 genomes in year 2015 with a total file size of 3TB. Medical Genome Reference Bank [4] is another whole genome sequencing database where the genomic data of 4000 Australia patients are stored. Research on other species such as the 3000 rice genomes project [5], giant salamander genome sequencing [6], the Arabidopsis thaliana project [7] also generated gigabytes or terabytes databases. Currently, the most ambitious project is the 100,000 Genomes Project, which plans to obtain 100,000 patients’ genome data for precision medicine research on cancer (https://www.genomicsengland.co.uk/the-100000-genomes-project). The increasing size of NGS databases has aroused significant interests and challenges in data analysis, storage and transmission. High-performance compression of genome databases is an effective way to address all of these issues.

Reference-based genome compression for compressing a single genome sequence has been intensively studied and achieved much higher compression ratio than reference free compression [8]. Existing reference-based genome compression algorithms include GDC [9], GDC2 [10], iDoComp [11], ERGC [12], HiRGC [13], CoGI [14], RlZAP [15], MSC [16], RCC [17], NRGC [18], SCCG [19] and FRESCO [20]. A straightforward application of these reference-based compression algorithms to solve the challenging problem of compressing a database containing *n* number of genome sequences is to conduct a one-by-one sequential reference-based compression for every genome in the database using one fixed reference genome.

A critical issue of this straightforward approach is the performance variation—the performance of reference-based algorithms highly depends on the similarity between the target and reference sequence, which can cause non-trivial performance variation in the compression of the same target sequence when a different reference is used. For instance, in a set of eight genome sequences, the compression ratios for genome hg19 by GDC2 [10] using seven different reference genomes varied remarkably from 51.90 to 707.77 folds [13]. Therefore, clustering similar genomes and specific reference identification within the clusters are of great significance in the compression of large scale genome databases.

We propose ECC, an **E**fficient **C**lustering-based reference selection algorithm for the **C**ompression of genome databases. Instead of using a fixed reference sequence by the literature methods, our idea is to cluster the genome sequences of the database into subsets such that genomes within one subset are more similar than the genomes in the other subsets, and then select the centroid genome as reference within each cluster for the compression. Then select a final reference to compress remaining centroid sequences.

We use the MinHash technique [21, 22] to measure the distance between sequences to construct a distances matrix of the genomes for the clustering. For a genomic sequence *L* (e.g., a chromosome sequence), MinHash first generates the set of constituent *k*-mers of *L*. Then the *k*-mers are mapped to distinct hash values through a hash function *H* (the set of hash values is denoted by *H*(*L*)). Then a small *q* number of the minimal hash values are sorted. This set of *q* smallest hash values is called a *sketch* of *H*(*L*) [22], denoted by *Sk*(*H*(*L*)). So, MinHash can map a long sequence (or a sequence set) to a reduced representation of *k*-mers which is called a sketch. Given two long sequences *L*_1_ and *L*_2_, MinHash uses some set operations on the sketches of *L*_1_ and *L*_2_ to efficiently estimate the distance between the original *L*_1_ and *L*_2_ under some error bounds. Recent studies have shown that sketch distance and MinHash are very effective in clustering similar genomic sequences with wide applications to genome assembly [23], metagenomics clustering [24], and species identification of whole genome sequences [22].

The main steps of our ECC method are as follows:

1. Construct a distance matrix of the *n* genome sequencesusing the pairwise sketch distance method Mash [22].
2. Utilize unsupervised learning to cluster the genomes based on the distance matrix, determine one reference sequence within each cluster and take the remaining ones as target sequences.
3. Compress the target sequences within each cluster by a reference-based compression algorithm, and a final reference sequence is selected for the compression of the remaining reference sequences.

The key differences between ECC and other compression schemes for sequence databases such as MSC [16] and RCC [17] include: (i) Our estimation on pairwise sequence distances is based on the sketch distance of the reduced *k*-mer sets [21] instead of the Euclidean distance between vectors of *k*-mer frequencies [17]; (ii) Our initial setting of the centroid in the clustering is not randomly as by RCC, but determined by the analysis on the whole database;(iii) The reference selection within the clusters is also decided by the clustering method instead of the reconstruction of the original target genome set by RCC.

The first difference implies that our approach is faster than the other methods and makes the clustering applicable to large sequence sets (RCC or MSC is limited to only short genome sequences due to its extremely high computational complexity). The second point of difference prevents the convergence to a local minimum for the **K**-medoids clustering method and makes the clustering results stable. The third point implies that our method compresses sequence set without the need to record additional information in the result. GDC2 is so far the best reference-based algorithm for the compression of the Human 1000 Genomes Database, the reference was selected external to the database. However, when the user is unfamiliar with the similarity between sequences in given set, the selection of one fixed reference sequence may result in very poor performance on dissimilar target sequences and a long running time in the compression. While the reference selection by ECC is decided by the clustering step, and all the reference are internal genomes of the database which are required to be compressed.

More related work in detail are provided in the next section to highlight the novelty of our method. In the experiments, we compared the performance on genome databases between the straightforward reference-fixed compression approach and our clustering approach ECC for the state-of-the-art reference-based compression algorithms. Our approach achieved 22.05% compression gain against the best case of the reference-fixed compression approach on a set of 60 human genomes collected from NCBI, where the compression ratio increases from 351.74 folds to 443.51 folds. On the union set of the Human 1000 Genomes Project and the 60-genome NCBI dataset, the compression ratio increases from 2919.58 folds to 3033.84 folds. Similar performance improvement over the rice genome database has also been observed.

## Related works

The assembled whole genome sequencing data are in the FASTA format. FASTA format is a text-based format for storing nucleotide data developed for biological sequence comparison [25]. It contains an identifier and multiple lines of sequence data. The identifier starts with greater symbol “ >”. The sequence data is constructed by the standard IUB/IUPAC code (International union of biochemistry, International Union of Pure and Applied Chemistry) [26] nucleic acids in base pairs represented using single-letter codes.

The common idea of the existing reference-based genome compression algorithms is to map subsequences in the target genome sequence to the reference genome sequence [8]. Firstly, an index such as a hash table or a suffix array is constructed from the reference genome to reduce the time complexity of the search process. Then an encoding strategy such as LZ77 [27] is applied to parse the target sequence to position number and length of the subsequence with regard to the reference sequence or mismatched subsequence. For instance, a subsequence in the target sequence is encoded as “102 72”, which stands for that this subsequence is identical to the subsequence from position 102 to 173 in the reference genome.

For a set of target genome sequences, the similarity between the reference sequence and the selected target sequence has a large effect on compression ratio. Existing attempts for reference selection in the compression of genome sequence databases can be categorized into three types. The first category selects a single reference genome to perform one-by-one sequential reference-based compression on all target genomes, which is named straightforward reference-fixed approach as in the previous section. Most of the reference-based compression algorithms applied that on genome set compression and select the single reference sequence randomly from the genome database, such as HiRGC [13], GECO [28], ERGC [12], iDoComp [11], CoGI [14], RLZ-opt [29], RLZAP [15]. GDC [9] and FRESCO [20] selects one single reference with a heuristic technique and provides fast random access. MRSCI [30] proposed a compression strategy that splits string set into references set and to-be-compressed set and then applied a multi-level reference-based compression.

The second category of algorithms utilizes not only one fixed reference for the compression of all sequences, but also the inter-similarity of the whole sequence set. Then it parses the subsequences not only based on the initial references but also the recorded pair. In other words, it considers all the compressed sequences as a ‘potential reference’ for the current compression. GDC2 [10] applies a two-level Ziv Lempel factorization [27] to compress large set of genome sequences. MSC [16] utilizes both intra-sequence and inter-sequence similarities for compression via searching subsequence matches in reference sequence and other parts of the target sequence itself, the compression order is determined by a recursive full search algorithm.

The third category of algorithms selects reference via unsupervised learning. RCC [17] performs clustering on the local histogram of dataset and derives a representative sequence of each cluster as the reference sequence for the corresponding cluster. A final representative sequence is then selected from the representative sequence set. For each cluster, the sequence data is compressed based on intra-similarity and inter-similarity with reference to the corresponding representative sequence. However, the derivation of representative sequence requires a large amount of time for assembly. The computation time is proportional to (*N*^2^*L*+*L*^2^), where *N* is the number of sequences and *L* is the average length of sequences. Hence it is not suitable for large-scale databases. In real experiment, it could not work on human or rice genome sequence set.

## Method

**Fig. 1:**
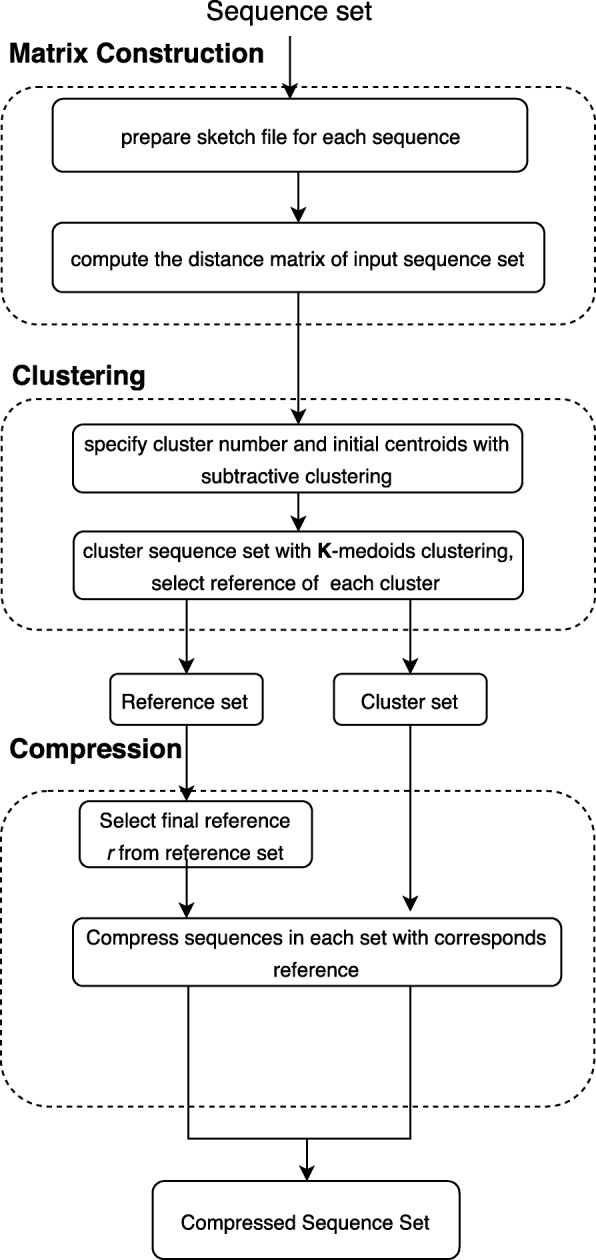
Schematic diagram of our algorithm ECC

Our algorithm ECC consists of three stages: Distance matrix construction for chromosome sequences, chromosome sequences clustering and chromosome sequences compression. A schematic diagram of the method is shown in Fig. 1.

### Construction of distance matrix for a set of chromosome sequences

Let $\mathcal {S} = \{S_{1}, S_{2}, \cdots, S_{n}\}$ be a collection of genomic sequences (i.e., a genome database or a chromosome database). We use a MinHash toolkit called Mash [22] to compute pairwise sketch distances of the sequences to form a distance matrix. By the tool Mash, a sequence *S*_*i*_ is firstly transformed into the set of its constituent *k*-mers, then all the *k*-mers are mapped to distinct 32-bit or 64-bit hash values by a hash function. Denote the hash values set of the constituent *k*-mers set from *S*_*i*_ as *H*(*S*_*i*_), and denote the set of *q* minimal hash values as *Sk*(*H*(*S*_*i*_),*q*), which is a size-reduced representative of *H*(*S*_*i*_), and is called a sketch of *H*(*S*_*i*_). For two hash-value sets *A* and *B*, the Jaccard index of*A* and *B* is defined as $J(A,B) = \frac {|A \cap B|}{|A \cup B|}$, and it can be estimated by $J^{\prime }(A,B) = \frac {|Sk(A \cup B,q) \cap Sk(A,q) \cap Sk(B,q)|}{|Sk(A\cup B,q)|}$. The sketch distance *d*_*sk*_ between two sequences *S*_*i*_ and *S*_*j*_ is defined as

1 $$ d_{sk}(S_{i}, S_{j}) = -\frac{1}{k}\ln{\frac{2*J^{\prime}(H(S_{i}),H(S_{j})) }{1+J^{\prime}(H(S_{i}),H(S_{j}))}}  $$

where the Jaccard index between *S*_*i*_ and *S*_*j*_ is approximately computed using the sketches of *H*(*S*_*i*_) and *H*(*S*_*j*_). We construct a distance matrix *M* for sequence set $\mathcal {S}$ with size *n*. *M* is a square matrix with dimension *n*×*n* that contains all the pairwise sketch distances between these genomic sequences. The elements of *M* are defined as:

2 $$ \begin{aligned} M_{ij} = \left\{\begin{array}{lc} 0\qquad \qquad \qquad \qquad \quad i=j \\ d_{sk}(S_{i}, S_{j}) \qquad \qquad \quad i \neq j \\ \end{array}\right. \\ i,j \in [1,n] \end{aligned}  $$

It is clear that *M* is a symmetric matrix (i.e., *M*_*ij*_=*M*_*ji*_). It can also be understood that the calculation of the sketch distance between two long sequences is much more efficient than the calculation by using *k*-mer feature vector direct comparison. The efficiency becomes significant, especially in the construction of the whole distance matrix *M*.

### Clustering of chromosomes from the distance matrix

Clustering is the process of grouping a set of samples into a number of subgroups such that similar samples are placed in the same subgroup. Here our clustering is to ensure a higher similarity between each reference-target pair for achieving an outstanding compression performance. An important step in the process of clustering is to determine the number of clusters in the data. We take a subtractive clustering approach [31, 32] to decide the number of clusters in the distance matrix *M*, and then use the **K**-medoids clustering method [33] to group the *n* number of genomic sequences into **K** number of clusters.

#### Subtractive clustering to determine the number of clusters **K**

Most clustering algorithms require the number of clusters as a parameter. However, the cluster number for a set of genomic sequences is normally unknown. We utilize a modified subtractive clustering algorithm to specify the cluster number.

Subtractive clustering is an extension of the Mountain method [34]. It estimates cluster centroid based on the density of points in the data space. We apply the exponential function for the Mountain Value Calculation. Given a sequence set $\mathcal {S}$, the corresponding sketch distance matrix *M* with dimension *n*×*n* and a threshold percentage *ε*∈(0,1), the process to determine the number of clusters is:

1. Create the empty cluster centroid set $\mathcal {O}$. Compute the mountain value of each sample *S*_*i*_:
  $Mt(S_{i}) = \sum _{j=1}^{n} e^{-M_{ij}}$
2. Let $o= \text {argmax}_{i=1}^{n} Mt(S_{i})$, add *S*_*o*_ to $\mathcal {O}$.
3. Update the mountain value of each remaining sequence by:
  $\phantom {\dot {i}\!}Mt(S_{i}) = Mt(S_{i}) - e^{-M_{io}}$
4. Repeat step 2 and 3 until *Mt*(*S*_*i*_)<*εMt*_*max*_ or $|\mathcal {O}|\geq \sqrt {n}$.
5. Return centroids set $\mathcal {O}$ and cluster number **K**$=|\mathcal {O}|$.

#### K-medoids clustering of the collection of *n* genomic sequences

**K**-medoids is a partition-based cluster analysis method. **K**-medoids iteratively finds the **K** centroids and assigns every sample to its nearest centroid [33], which is similar to **K**-means [35] but more effective for handling outliers. It divides the data set $\mathcal {S}$ into **K** non-overlapping subgroups $\mathcal {C}$ that contains every element of $\mathcal {S}$ and select a centroid sequence *O*_*i*_ from each subgroup:

##### **Definition 1**

For a set of sequence $\mathcal {S} = \{S_{1},\cdots,S_{n}\}$, the corresponding cluster set $\mathcal {C}=\{C_{1},C_{2},\cdots,C_{K}\}$ and centroid sequence set $\mathcal {O}=\{O_{1},O_{2},\cdots,O_{K}\}$ satisfies the following requirements:$C_{i} \subseteq \mathcal {S},C_{1} \cup C_{2} \cup \cdots \cup C_{K} = \mathcal {S},C_{i} \cap C_{j} = \emptyset $ for *i*≠*j*, *O*_*i*_∈*C*_*i*_.

The cluster set $\mathcal {C}$ is determined via minimizing the cost function *λ* as follows:

$$\lambda(\mathcal{S}) = \sum_{i=1}^{K}\sum_{S_{a} \in C_{i}}d_{sk}({S}_{a},{O}_{i}) $$

Though **K**-medoids is efficient, it has some drawbacks. The clustering result highly depends on the setting of the initial centroids. To improve the stability and quality of clustering result, instead of arbitrarily selecting the initial centroids by the standard **K**-medoids, we use the centroid set $\mathcal {O}$ as computed by subtractive clustering in previous section.

Given a sequence set $\mathcal {S}$, sketch distance matrix *M*, cluster number **K** and centroid sequence set $\mathcal {O}$, the **K**-medoids proceeds by the following steps:

1. Set $\mathcal {O}$ as the initial centroid sequence set.
2. Associate each *S*_*i*_ to the centroid *O*_*j*_ with minimum sketch distance, also associate *S*_*i*_ to cluster *C*_*j*_.
3. Recalculate the new centroid of each cluster based on its elements :
  $$O_{j} = \underset{S_{a} \in C_{j}}{\text{argmin}} \sum_{S_{b} \in C_{j}}d_{sk}(S_{a},S_{b}) $$
4. Repeat steps 2 and 3 until $\mathcal {C}$ and $\mathcal {O}$ no longer change or reach a pre-set number of iterations.
5. Return cluster set $\mathcal {C}$ and cluster centroid set $\mathcal {O}$.

### Compression

Chromosome sequences set $\mathcal {S}$ is compressed based on the cluster set $\mathcal {C}$ and centroids set $\mathcal {O}$ computed by **K**-medoids. First, use *O*_*i*_ as the reference sequence for the other sequences in cluster *C*_*i*_. Then select a final reference *R* from the centroid set as the reference for the other centroid sequences:

$$r= \underset{O_{i} \in \mathcal{O}}{\text{argmin}} \sum_{O_{j} \in \mathcal{O}}d_{sk}(O_{i},O_{j}) $$

In detail, all the sequences in cluster *C*_*i*_ is compressed using *O*_*i*_ as the reference sequence except *O*_*i*_ itself. Then all the reference sequences except *R* is compressed using *R* as the reference sequence. The final reference *R* can be compressed by the block-sorting compression (bsc) algorithm (http://libbsc.com/) or other reference-free compression algorithms.

All non-centroids sequences will be compressed with centroid sequences as reference and centroid sequences (except *R*) will be compressed with *R* as reference, only one final reference sequence *R* will remain uncompressed. It is clear that the same number of sequences is compressed in ECC as in straightforward approach.

All reference-based compression algorithms can take this clustering approach to compress a set of genomic sequences. The pseudo-code of our compression method is presented in Algorithm 1.


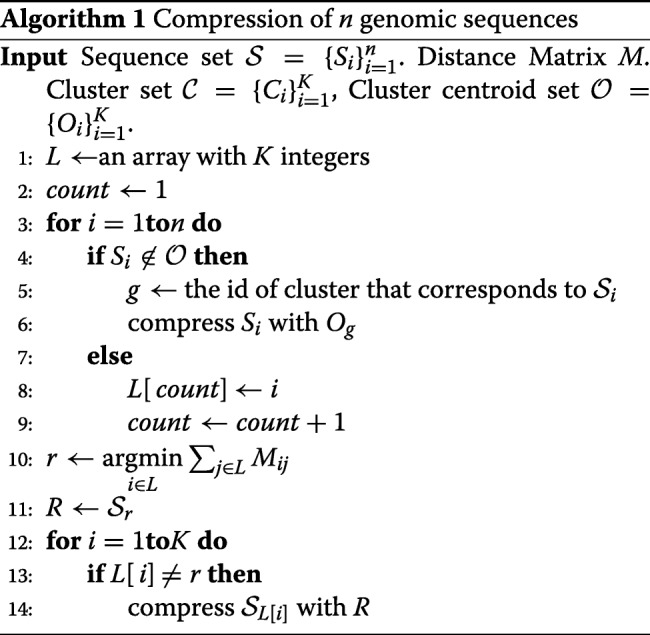


### Decompression

The decompression process is the reversion process of compression. All the sequences except *R* require a reference to decompress. Firstly, *R* is decompressed; then the reference sequence of each cluster is decompressed by *R*, all the remaining sequences in the cluster are decompressed by the reference sequence in its cluster. As the process is invertible, the compression scheme is lossless as long as the used reference-based compression algorithm is lossless.

### Data

To assess the performance of our proposed method ECC, we compare the compression ratio based on ECC result with the reference-fixed compression approach on multiple genome databases.

These include: a set of 60 human genome sequences (denoted by dataset-60) from National Center for Biotechnology Information (NCBI) with a file size of 171 GB, a set of 1152 human genome sequences (dataset-1152) from the 1000 Genomes Project [2] and NCBI having a file size of 3128 GB, and a set of 2818 rice genomes (dataset-2818) from the 3000-rice project [36] having a file size of 1012 GB.

## Results and discussion

This section describes our experimental results on dataset-60, dataset-1152 and dataset-2818 to evaluate the performance of our approach. In particular, the compression ratio and running time of our algorithm are presented and discussed in comparison with the reference-fixed compression approach.

### Test methodology

Our algorithm was implemented in the C++11 language. All experiments were conducted on a machine running Red Hat Enterprise Linux 6.7 (64 bit) with 2 × Intel Xeon E5-2695 processors(2.3GHz,14 Cores), 128 GB of RAM, and 4 cores.

Six state-of-the-art reference-based compression algorithms were tested on the three genome databases to understand the performance improvement achieved by our clustering approach in comparison with the reference-fixed compression approach. These compression algorithms are HiRGC [13], iDoComp [11], GDC2 [10], ERGC [12],NRGC [18] and SCCG [19]. All the algorithms that are compatible with multi-cores computing were executed with 4 cores.

We also attempted to test the performance of RCC [17] on the same genome databases. However, it was not runnable for the compression of long genome sequences (such as human and rice) due to its time complexity— RCC was taking longer than 10 h to compress only four human genome sequences.

For GDC2, as its two-level compression structure tends to compress all the target sequences using the same reference, we compress the datasets using the final reference selected by ECC, and the compression order of GDC2 is also adjusted in accordance with the ECC clustering result.

As mentioned before, the performance of a reference-based algorithm on the NGS dataset is highly dependable on the option of the reference sequence. To reduce the variance from an arbitrary selection, we randomly selected multiple reference sequences from the target dataset and obtain the compression performance with each of them for the compression algorithms (the randomly selected reference file itself is not compressed, so all experiments compress the same number of genome sequences).

To measure the performance improvement, we denote the compression ratio with fixed single reference as *C*_*S*_ and the compression ratio on same dataset with ECC as *C*_*E*_, and introduce a relative compression ratio gain as:

$$G = \left(1 - \frac{C_{S}}{C_{E}}\right)\times 100\% $$

A larger value of compression ratio gain indicates a more significant improvement. Due to page limitation, we only report the compression gain against the **best** result of the reference-fixed compression approach for the reference-based compression methods.

### Gains of compression performance

Our proposed ECC method outperforms over the reference-fixed compression approach in all cases on dataset-60 (see Table 1). The compression gains against the best results by the reference-fixed compression approach are 22.05%, 22.83%, 2.22%, 56.31%, 3.41%, 15.49% for HiRGC, iDoComp, GDC2, ERGC, NRGC, and SCCG respectively. On dataset-60, HiRGC, iDoComp, ERGC and SCCG gained more compression improvement, while the effect of ECC on NRGC and GDC2 is relatively smaller. Moreover, HiRGC, iDoComp, SCCG and GDC2 achieved higher compression ratio on this database than ERGC and NRGC in general.

**Table 1:** Compression ratio for the H. sapiens dataset-60 (171GB)

Reference	Compression ratio with algorithm					
	HiRGC	iDoComp	GDC2	ERGC	NRGC	SCCG
GCA_000004845	339.80	*184.20*	238.98	11.00	122.67	225.35
hg19	346.80	26.78	*242.60*	*128.11*	137.41	*265.03*
YH	*351.74*	134.13	237.39	108.26	123.24	228.20
GCA_000252825	241.01	92.65	230.45	102.62	176.08	122.25
Huref	245.79	140.84	224.47	69.59	*177.85*	123.26
ECC clustering result	**443.51**	**238.68**	**248.11**	**293.24**	**184.13**	**313.60**
Ratio gain*	22.05%	22.83%	2.22%	56.31%	3.41%	15.49%

Bold text indicates the highest compression ratio of an algorithm, italic text indicates the **best** case of fixed single reference compression result

*The ratio gain of ECC against the **best** case

We added the 1092 human genomes from the 1000 Genome Project to dataset-60 (denoted by H. sapiens dataset-1152) and conducted another round of experiments. Performance details are summarized in Table 2 for HiRGC, iDoComp and GDC2 which are the three algorithms of the highest compression performance on dataset-60. The overall compression performance is higher than on dataset-60. Through ECC, iDoComp gained 15.86% compression performance against the best reference-fixed compression case, while HiRGC gained 7.95%. The ratio gain of GDC2 is only 3.77%, but more importantly, ECC helped GDC2 avoid 3 of the 7 time-consuming cases in the reference-fixed approach.

**Table 2:** Compression ratios on H. sapiens dataset-1152 (3128 GB)

Reference	Compression ratio with algorithm		
	HiRGC	iDoComp	GDC2
HG00096	991.77	*485.35*	*2919.58*
NA18856	889.32	437.05	2805.19
GCA_000004845	784.84	53.94	2901.44
GCA_000252825	504.41	114.40	2897.76
GCA_000365445	13.07	/	/
hg19	*1046.84*	68.36	/
hg38	826.31	52.03	/
Result of ECC	**1137.21**	576.84	3033.84
Ratio gain*	7.95%	15.86%	3.77%

’/’ indicates a running time longer than 500 h. Bold text indicates the highest compression ratio of an algorithm, italic text indicates the **best** case of fixed single reference compression result.

*The ratio gain of ECC against the **best** case

On the rice genome dataset-2818, through our ECC clustering approach, HiRGC gained 13.89% compression performance against the best case by the reference-fixed compression approach, iDoComp gained 21.22%, and GDC2 gained 2.48% (Table 3). The compression ratio gain of HiRGC is more stable than on the first two human genome databases. A reason is that all the genomes in the rice database were aligned to the sequenced rice cultivars: 93-11 (indica variety) [37]. Hence this dataset has a higher inter-similarity and the variance from the random selection of the fixed reference is smaller.

**Table 3:** Compression ratio on the Oryza sativa Ldataset-2818(1012 GB)

Reference	Compression ratio with algorithm		
	HiRGC	iDoComp	GDC2
B035	*79.31*	77.62	529.40
CX319	73.76	*81.55*	*537.09*
IRIS_313-10010	69.93	64.74	519.43
IRIS_313-10776	70.97	77.10	533.81
IRIS_313-9937	71.39	66.42	535.31
Result of ECC	**92.10**	**103.52**	**550.77**
Ratio gain*	13.89%	21.22%	2.48%

Bold text indicates the highest compression ratio of an algorithm, italic text indicates the **best** case of fixed single reference compression result

*The ratio gain of ECC against the **best** case

From these comparisons, we can understand that our ECC clustering approach can make significant compression improvement for most of the state-of-the-art algorithms and can avoid selecting some inappropriate references such as the 3 extremely time-consuming cases of GDC2 on the human dataset-1152.

### Speed performance

Running time is an essential factor for measuring the applicability of an algorithm in the compression of large-scale genome databases.The running time of ECC includes two parts: reference selection time (only depending on the input sequence set) and the compression time (depending on the input sequence set and the reference-based compression algorithm). The detailed compression time of each reference-based compression algorithm with difference references are listed in Additional file 1.

As shown in Table 4, ECC took 0.02, 0.83, 0.76 h on the reference selection part for dataset-60, dataset-1152 and rice genome dataset-2818 respectively. But the compression time for these three datasets are 0.98, 13.94, 2.82 h (Table 5) by HiRGC,which is the fastest algorithm in the compression. The reference selection time is much shorter than the sequence compression time.

**Table 4:** Reference selection time of ECC (in hours)

Dataset	dataset-60	dataset-1152	dataset-2818
Number of genomes	60	1152	2818
Total running time	0.023	0.830	0.759

**Table 5:** Compression time of each algorithm on the three datasets

Algorithm	Compression time (in hours) for					
	dataset-60		dataset-1152		datset-2818	
	reference-fixed	ECC	reference-fixed	ECC	reference-fixed	ECC
HiRGC	1.18	0.98	15.12	13.94	2.91	2.82
iDoComp	6.54	2.82	102.94	29.77	15.58	10.34
GDC2	110.73	117.82	129.24	126.43	25.29	23.61

The time by the reference-fixed approach is the average running time of several fixed single-reference cases by each algorithm, please see the supplementary file for the time range of all the cases and compression time by ERGC, SCCG and NRGC

We have also observed that the total time of reference selection and compression by ECC is highly competitive with the reference-fixed compression approach. In fact, the compression time via ECC after the reference selection is shorter than the compression time of the reference-fixed compression in most cases except GDC2 on the dataset-1152 (Table 5).

## Conclusion

In this work, we introduced ECC, a clustering-based reference selection method for the compression of genome databases. The key idea of this method is the calculation of a MinHash sketch distance between chromosome sequences to group the chromosome sequences into subsets of similar sequences. Within each cluster, the reference chromosome is best updated according to the shortest sketch distance to the centroid chromosome. This algorithm is universal for genome sequence sets of the same species. We have demonstrated that the six state-of-the-art reference-based compression algorithms all achieved a substantial improvement after the clustering of the genome sequences, with similar amounts of compression time consumed by the reference-fixed approach.

Although ECC provides an efficient reference selection scheme for reference-based compression, there are some other aspects that are worth consideration for further improvement. First, ECC is unable to handle dynamic genome sequence dataset. When new sequence added to compressed dataset, it can only be compressed with the final reference in previous. There are two possible ways to solve that: 1. Store the sketch set information of existing centroid sequences and update the clustering result based on new sequence. 2. Select the reference for new sequence via heuristic method. In addition, we did not exploit the structure of representative sequences of each dataset provided. If make full use of the *k*-mer features computed in distance matrix construction stage, it is possible to construct a universal sequence via merging *k*-mers with suffix-prefix overlaps. There are some research works proposed for merging sequence with suffix-prefix overlaps [38]. We will investigate these issues to provide new functionalities on top of current ECC.

## Supplementary information

**Additional file 1** The list of researched dataset and supplementary results

- The list of genomes in 60-dataset.
- The access to dataset from 1000 Genomes Project and 3000 rice genomes project.
- The compression time(in hours) for algorithms using different references on three datasets.
- Compressed size of chromosome 1 of dataset-60 with different cluster number *K*.

## Data Availability

All data associated with this study are available in the Supplementary Data file under Additional Files. The C++ codes of our algorithm are available at https://github.com/Tao-Tang/ECC.
