# Supplementary material for: Sketch distance-based clustering of chromosomes for large genome database compression
Source: BMC Genomics. 2019 Dec 30;20(Suppl 10):978. doi: 10.1186/s12864-019-6310-0 (PMC6939838; doi:10.1186/s12864-019-6310-0)
Supplement: Supplementary file 1 — Additional file 1 The list of researched dataset and supplementary results [file 12864_2019_6310_MOESM1_ESM.pdf]

Sketch distance-based clustering of chromosomes for large genome database compression

Tao Tang¹, Yuansheng Liu¹, Buzhong Zhang², Benyue Su², and Jinyan Li^{1,*}

¹Advanced Analytics Institute, University of Technology Sydney, Broadway, NSW 2007, Australia

²School of Computer and Information, Anqing Normal University, Anqing 246401, China

*Corresponding Author

October 9, 2019

Table S1: List of genomes in 60-dataset (H. sapiens, 171GB)

GenBank assembly accession of genomes from NCBI				
GCA_000004845	GCA_003112955	GCA_003113175	GCA_003112915	GCA_003113115
GCA_001750385	GCA_001007805	GCA_001421375	GCA_003112975	GCA_002180035
GCA_000212995	GCA_001856745	GCA_001015355	GCA_001549595	GCA_000002115
GCA_001500185	GCA_002009925	GCA_001420765	GCA_003113215	GCA_001292825
GCA_000365445	GCA_002077035	GCA_003112855	GCA_000772585	GCA_001524155
GCA_001708065	GCA_003113235	GCA_002022525	GCA_003112815	GCA_001712695
GCA_003112935	GCA_003112835	GCA_000306695	GCA_003112895	GCA_001483585
GCF_000002125	GCA_001549605	GCA_001307025	GCA_001542345	GCA_002008905
GCA_001483655	GCA_001307015	GCA_001483605	GCA_003112875	GCA_001015385
GCA_000005465	GCA_000005465	GCF_000001405	GCA_000983455	GCA_000001405
GCA_001307125	GCA_002208065	GCA_000252825.1_HuRef	YH	Huref
hg19	hg38	K224	K131	GCA_000252825.1_HuRefPrime

Dataset from 1000 Genomes Project

The 1000 Genomes Project contains information of 1092 person, 525 males and 567 females. The VCF files can be downloaded from:

`ftp://ftp.ncbi.nih.gov//1000genomes/ftp/phase1/analysis_results/integrated_call_sets/{FILENAME}`

Dataset from 3,000 rice genomes project

The list of rice genomes from 3000 rice genomes project is available at:

`https://irods-webdav.asti.dost.gov.ph/pub/3kRG/nipponbare/nipponbarevcf_manifest.txt`

To download specific file, use:

`wget --no-check-certificate https://anonymous:anonymous@irods-webdav.asti.dost.gov.ph/pub/FILENAME`

Table S2: Compression time (in hours) for algorithms with different references on dataset-60

Algorithm	GCA_000004845	hg19	YH	GCA_000252825	Huref	result of ECC
HiRGC	1.05	0.96	1.13	1.29	1.45	0.98
iDoComp	3.28	12.04	4.52	7.23	5.62	2.82
GDC2	90.10	163.02	87.31	105.83	107.38	117.82
ERGC	31.30	30.68	32.56	27.86	32.47	30.83
NRGC	10.68	18.57	14.76	13.65	6.98	13.07
SCCG	26.52	24.30	24.79	24.82	31.75	20.78

Table S3: Compression time (in hours) for algorithms with different references on dataset-1152

Algorithm	<i>RA</i>	<i>RB</i>	<i>RC</i>	<i>RD</i>	<i>RE</i>	<i>RF</i>	<i>RG</i>	ECC
HiRGC	13.07	19.98	11.66	12.54	23.42	11.85	13.30	13.94
iDoComp	27.25	143.06	235.17	94.11	187.41	17.83	15.76	29.77
GDC2	103.57	221.07	89.01	103.30	/	/	/	126.43

*RA:GCA_000004845, *RB*:GCA_000252825, *RC*: HG00096, *RD*: NA18856,
RE:GCA_000365445, *RF*:hg19, *RG*:hg38

Table S4: Compression time (in hours) for algorithms with different references on dataset-2818

Algorithm	B035	CX319	IRIS_313-10010	IRIS_313-10776	IRIS_313-9937	ECC
HiRGC	2.98	2.90	2.87	2.86	2.92	2.82
iDoComp	16.42	16.63	18.29	13.52	13.02	10.34
GDC2	21.17	24.14	27.81	26.47	26.84	23.61

Compressed size of chromosome 1 of dataset-60 with different cluster number K

In standard ECC method, cluster number K is determined by subtractive clustering, then we set K as fixed value without using subtractive clustering to verify the best K values for compression.

As shown in figure 1 and table 5, the K value determined by subtractive clustering achieved the smallest compressed size, apart from subtractive clustering, $K = 10$ outperforms other fixed values.

Table S5: Compressed size of chromosome 1 of dataset-60 with different K values

K	2	4	6	8	10	Subtractive clustering
Compressed size	38225920	36956160	36966400	37949440	35266560	34928640

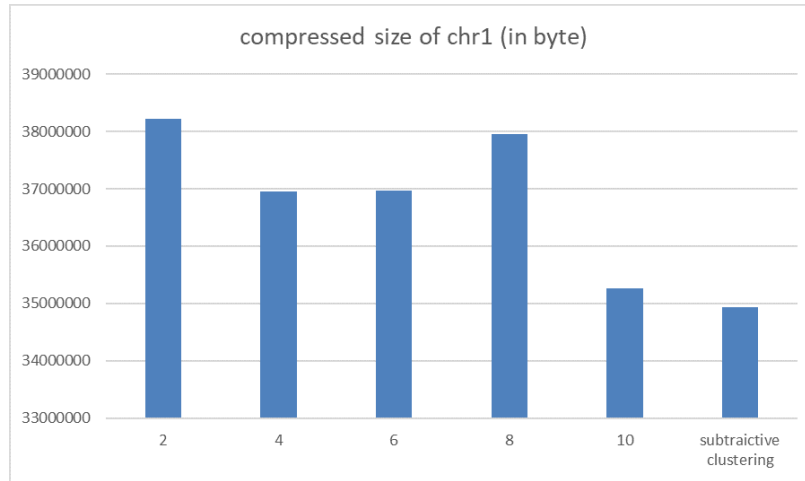

Figure S1: Compressed size of chromosome 1 of dataset-60 with different K values
